# Zika virus infection and microcephaly: Evidence regarding geospatial associations

**DOI:** 10.1371/journal.pntd.0006392

**Published:** 2018-04-25

**Authors:** João Ricardo Nickenig Vissoci, Thiago Augusto Hernandes Rocha, Núbia Cristina da Silva, Rejane Christine de Sousa Queiroz, Erika Bárbara Abreu Fonseca Thomaz, Pedro Vasconcelos Maia Amaral, Adriana Lein, Maria dos Remédios Freitas Carvalho Branco, José Aquino, Zulimar Márita Ribeiro Rodrigues, Antônio Augusto Moura da Silva, Catherine Staton

**Affiliations:** 1 Duke Global Health Institute, Duke University, Durham, North Carolina, United States of America; 2 Department of Surgery, Division of Emergency Medicine, Duke University Health System, Durham, North Carolina, United States of America; 3 Federal University of Minas Gerais, School of Economics, Center of post-graduate and Research in Administration, Belo Horizonte, Minas Gerais, Brazil; 4 Federal University of Minas Gerais, Faculty of Economics, Observatory of Human Resources in Health, Belo Horizonte, Minas Gerais, Brazil; 5 Federal University of Maranhão, Department of Public Health, São Luís, Maranhão, Brazil; 6 Federal University of Minas Gerais, Centre for Development and Regional Planning, Belo Horizonte, Minas Gerais, Brazil; 7 Federal University of Maranhão, Department of Pathology, São Luís, Maranhão, Brazil; 8 Federal University of Maranhão, Department of Geosciences, São Luís, Maranhão, Brazil; University of Heidelberg, GERMANY

## Abstract

**Background:**

Although the Zika virus (ZIKV) epidemic ceased to be a public health emergency by the end of 2016, studies to improve knowledge about this emerging disease are still needed, especially those investigating a causal relationship between ZIKV in pregnant women and microcephaly in neonates. However, there are still many challenges in describing the relationship between ZIKV and microcephaly. The few studies focusing on the epidemiological profile of ZIKV and its changes over time are largely limited to systematic reviews of case reports and dispersal mapping of ZIKV spread over time without quantitative methods to analyze patterns and their covariates. Since Brazil has been at the epicenter of the ZIKV epidemic, this study examines the geospatial association between ZIKV and microcephaly in Brazil.

**Methods:**

Our study is categorized as a retrospective, ecological study based on secondary databases. Data were obtained from January to December 2016, from the following data sources: Brazilian System for Epidemiological Surveillance, Disease Notification System, System for Specialized Management Support, and Brazilian Institute of Geography and Statistics. Data were aggregated by municipality. Incidence rates were estimated per 100,000 inhabitants. Analyses consisted of mapping the aggregated incidence rates of ZIKV and microcephaly, followed by a Getis-Ord-Gi spatial cluster analysis and a Bivariate Local Moran’s I analysis.

**Results:**

The incidence of ZIKV cases is changing the virus’s spatial pattern, shifting from Brazil’s Northeast region to the Midwest and North regions. The number of municipalities in clusters of microcephaly incidence is also shifting from the Northeast region to the Midwest and North, after a time lag is considered. Our findings suggest an increase in microcephaly incidence in the Midwest and North regions, associated with high levels of ZIKV infection months before.

**Conclusion:**

The greatest burden of microcephaly shifted from the Northeast to other Brazilian regions at the beginning of 2016. Brazil’s Midwest region experienced an increase in microcephaly incidence associated with ZIKV incidence. This finding highlights an association between an increase in ZIKV infection with a rise in microcephaly cases after approximately three months.

## Author summary

The increasing evidence of a relationship between ZIKV in pregnant women and fetal congenital ZIKV syndrome with microcephaly has been reported in the literature over the last two years. Our findings suggest a spatial dependency between the diseases. Therefore, using the spatial pattern of ZIKV incidence to better understand risk areas for microcephaly may help the design of surveillance policies. Brazil had a large epidemic of ZIKV, leading to several important studies of the ZIKV outbreak and its association with microcephaly. This study used a geospatial analysis approach to examine the association between ZIKV and microcephaly in Brazilian regions. It was possible to highlight a spatial association between ZIKV and microcephaly considering a time lag between diseases. Brazilian regions with the highest incidences of microcephaly were the regions where the highest incidence of ZIKV occurred months before. This finding can help the organization and planning of health services to offer better screening actions dedicated to pregnant women in high-risk areas.

## Introduction

On February 1, 2016, the World Health Organization (WHO) declared that the Zika virus (ZIKV) epidemic was an international public health emergency [1]. The increasing evidence of a causal relationship between ZIKV in pregnant women and an unpredicted rise in the incidence of microcephaly, later characterized as fetal congenital ZIKV syndrome [2–4], prompted this designation. Findings suggest that ZIKV affects neurogenesis during human brain development, leading to neurological syndromes as observed in Guillain-Barré or microcephaly [4]. As of the latest ZIKV status report in March 2017, 48 of 50 countries and territories in the Americas have confirmed autochthonous cases of ZIKV [5]. Half of these countries and territories (24) have confirmed cases of congenital ZIKV syndrome [5].

Brazil remains at the epicenter of the ZIKV epidemic with reports of 130,000 cases in 2016 [6]. In October 2015, early warning signs of a link between ZIKV in pregnant women and microcephaly in neonates surfaced when the number of infants born with microcephaly in the Northeastern state of Pernambuco rose [7]. From 2015 to 2016, 2,229 cases of microcephaly in infants were confirmed [8], over a 10-fold increase from the yearly average of 157 cases between 2000 and 2014 [9]. Consequently, a body of literature has emerged supporting a causal association between ZIKV infection during pregnancy and infant microcephaly [2,10–12]. Existing literature focused on the Brazilian ZIKV epidemic consists heavily of clinical management guidelines and longitudinal and case-control studies of mothers diagnosed with ZIKV and their infants to assess risks of congenital ZIKV syndrome [13,14].

Despite the ongoing research, challenges related to preventing ZIKV and its consequences, such as microcephaly, are still staggering. First, the committed countries have a limited epidemiological surveillance capacity. Second, the time delay between the onset of the ZIKV epidemic and the microcephaly reports means public policy is still defining the epidemic and not yet able to prevent its consequences. The rise in microcephaly incidence was documented only after infants were born, mostly due to limited ZIKV testing during intrapartum infection, leading to delays in timely epidemiologic and geographic surveillance of both diseases.

Using mapping techniques to study vector-borne disease epidemiology has proven crucial, as seen with previous research on dengue virus [15] and chikungunya [16]. These health geography studies can identify disease propagation patterns and high-risk areas, then model forecasts allowing inferences for the determinants of these outcomes [17]. To date, however, few studies have utilized geospatial techniques to investigate the ZIKV epidemic in Brazil, and the spatial-temporal association between ZIKV and microcephaly remains uninvestigated. The only available works [18,19] rely on systematic reviews of case reports or dispersal mapping of ZIKV spread over time without quantitative methods to analyze patterns and their covariates. By using a framework of health geography, we believe we can provide insights into disease spread patterns, high-risk areas, and forecast disease models that allow for inferences regarding the determinants of these outcomes [17].

This study examines the geospatial association between ZIKV and microcephaly January—December 2016. Specifically, we aim to 1) spatially represent diffusion patterns for both ZIKV and microcephaly incidence; 2) identify hot and cold spots of high and low incidence clusters for both diseases and any changes in their distribution across time; and 3) measure the spatial-temporal association between ZIKV and microcephaly spread. We hypothesize that areas with higher ZIKV incidence will be positively associated with an increase in microcephaly incidence after a time lapse of at least 16 weeks [9].

## Methods

### Study design, setting, and participants

This ecological, retrospective study utilizes secondary data analysis of national health data systems during the ZIKV epidemic from January to December 2016 in Brazil. The largest country in Latin America in both size and population, Brazil spans approximately 3.2 million square miles with an estimated 190.7 million inhabitants [20]. An upper middle—income country and member of BRIC, Brazil ranks ninth among global economies [21] and has a high human development index level of 0.754 [22].

**Fig 1:**
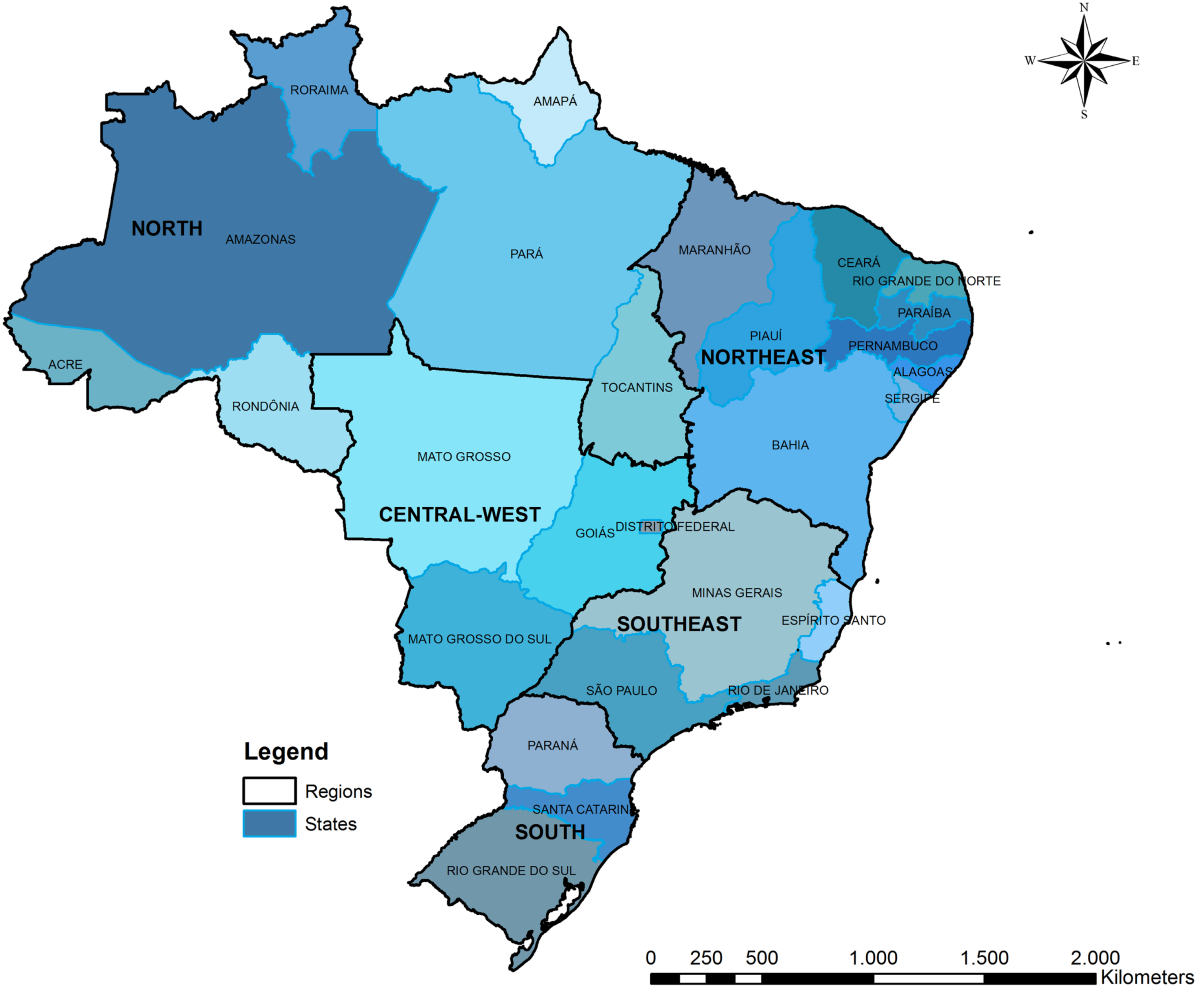
Brazilian states and regions.

Brazil achieved universal health care coverage in the 1990s with the implementation of and reforms to the Unified Health System (SUS) [23]. Driven by national policies favoring decentralization and community-based models of health services delivery, the structure of the SUS is conducive to ecological studies of health outcomes [24]. The SUS maintains over 15 national-level health informatics and epidemiological databases to guide population health surveillance [24]. Data of infrastructure to outcome indicators are available, comprising information at individuals, municipalities or states levels. [6]. Marked inequality among Brazil’s regions, namely lower development levels and widespread poverty in the Northeast, results in disparities in health services coverage and population health indicators [25], which are significant when addressing diseases with natural and built environmental determinants (Fig 1). The availability of publicly accessible government databases at the national level, coupled with the socio-geographic landscape of the country and manifestations of the ZIKV epidemic, make Brazil an optimal setting in which to investigate the spatial-temporal association between ZIKV infection and microcephaly spread.

### Data sources

Data on confirmed cases of ZIKV were obtained from the Disease Notification System [26]. ZIKV infection was included in the Brazilian Ministry of Health compulsory notification disease list on February 17, 2016. From this date on, every health system unit in Brazil was obligated to report any confirmed or suspected case of ZIKV to the Ministry of Health [27]. ZIKVnotification is performed on a weekly basis, and deaths related to ZIKV must be reported within a maximum of 24 hours of death. Notification information is uploaded to the SINAN NET system (acronym in Portuguese—Disease Notification Information System) [28]. During our study, we included only confirmed cases of ZIKV. A suspected case was considered confirmed if one of the following characteristics was observed: positive of viral isolation test result, RNA viral detection by reaction of reverse transcriptase, or IgM serology. After confirmation of autochthonous circulation, the cases of Zika should be confirmed by clinical-epidemiological criteria. Despite that, suspected cases in pregnant women, neurological manifestations, and death still need to be confirmed using a serology test [27].

Data on confirmed cases of microcephaly were retrieved online from the System for Specialized Management Support [8]. Microcephaly was defined as an infant with 37 or more weeks of gestation with a head circumference equal to or less than 31.9 cm for male infants, or equal to or less than 31.5 cm for female infants, in concurrence with WHO standards [1]. For babies less than 37 weeks gestation at birth, the InterGrowth curve was used since the cephalic perimeter varies according to an infant’s gestational age [1]. Monthly case reports of microcephaly must be sent to the RESP-Microcephaly system (acronym in Portuguese—Register of Events in Public Health for Microcephaly). Additionally, population data were obtained from the Brazilian Institute of Geography and Statistics (Instituto Brasileiro de Geografia e Estatística) [20]. Data from these three sources were merged for all 5570 Brazilian municipalities. All secondary data extracted correspond to 2016.

### Data analysis

Raw values of confirmed cases of ZIKV and microcephaly were used to compute incidence rates. Incidence rates were expressed continuously per 1,000 inhabitants for ZIKV and 100,000 for microcephaly, at the municipal level. We opted to use different scales because the prevalence of ZIKV and microcephaly in the general population occur on different scales. Ideally, we would have used better exposure controls such as pregnant women and newborns, such as the results reported by De Oliveira et al [29]. Thus, we decided to present the results in indices by population, which would give us a robust metric.

Data analyses were carried out in three steps. First, we conducted a descriptive analysis for the aggregated incidence rates of ZIKV and microcephaly for six 2-month time periods between January and December 2016 at the regional level. As such, the first bi-monthly period comprised January and February, the second period March and April, and so on. Next, we conducted a Getis-Ord-Gi [30] spatial cluster analysis to identify the presence of clustering according to incidence rates of both diseases throughout 2016. The Getis-Ord-Gi analysis produced two types of spatial clusters: hotspots with high values of incidence of both diseases, and coldspots highlighting low incidence areas. Lastly, a Bivariate Local Moran’s I analysis was carried out to evaluate the temporal-spatial association between ZIKV and microcephaly incidence rates over time [31].

The Bivariate Local Moran’s I is a statistic that evaluates the spatial correlation between two variables [31]. It verifies whether the value of the first variable in a reference municipality is related to the average value of the second variable in neighboring municipalities. Therefore, if the two variables are measured in different time periods, and a long enough time lapse is taken into account, this technique can provide insights regarding whether previous incidence of ZIKV infection in any reference municipality is associated with microcephaly cases in the neighboring region. This analytical strategy relies on the assumption that ZIKV has a causal role in microcephaly when pregnant women are infected [4,13]. Although there is not a consensus of the exact time in pregnancy that a ZIKV infection will cause microcephaly, there is a high volume of evidence supporting the association [14,32]. Thus, a time lag between ZIKV infection and microcephaly incidence can be approximated to the gestational period (in our case, 3 to 4 bi-monthly time periods) [9]. Therefore, considering the importance of a time lag between ZIKV and the emergence of microcephaly cases we opted to test multiple scenarios. For each scenario, a minimum difference of one bi-monthly period was considered. Incidence of ZIKV infection during the first and second bi-monthly periods were compared to microcephaly incidence rates of the third to sixth bi-monthly periods. This time arrangement was applied to all 2016 bi-monthly periods for both diseases, for all possible combinations that respect a minimum time lag of two bi-monthly periods. We chose this time lag period considering previous findings by the Centers for Disease Control [9].

The categorization provided by Bivariate Local Moran’s I technique can identify clusters based on ZIKV incidence considering, simultaneously, the microcephaly levels months later. In this scenario, a High-High cluster, for example, would represent a group of municipalities with elevated rates of microcephaly surrounded by municipalities with high values of ZIKV incidence a given number of months beforehand.

Incidence rate mapping and the Getis-Ord-Gi cluster analysis were performed in ARCGIS 10.3 [33]. The Bivariate Local Moran’s I analysis was conducted using the software GEODA [34].

## Results

From January—December 2016, Brazilian incidence rates of ZIKV per 100,000 inhabitants varied from 13.01 to 0.21. During the year, ZIKV incidence substantially decreased in all regions. Though this reduction was observed in all regions, it was more pronounced in the Midwest and Northeast regions. A high number of Midwest region municipalities showed incidence rates above 20 cases per 100,000 inhabitants during the first and second bi-monthly periods. At these time points, the Midwest contained the greatest number of confirmed ZIKV cases, with mean incidence rates of 82.06 for the first bi-monthly period and an annual average of 21.36 cases per 100,000 inhabitants. ZIKV incidence decreased in the following bi-monthly periods of the year. During the third bi-monthly period, the greatest mean ZIKV incidence rate was seen in the Northeast (7.56), which also had the greatest mean (2.81) for the fourth bi-monthly period. The fifth and sixth bi-monthly periods were marked by a continued reduction of high- and medium-incidence municipality clusters. By the fifth and sixth bi-monthly periods, mean ZIKV incidence rates had declined, with the highest for the fifth bi-monthly period in the Northeast (0.61) and the highest for the sixth bi-monthly period in Midwest (0.42).

**Fig 2:**
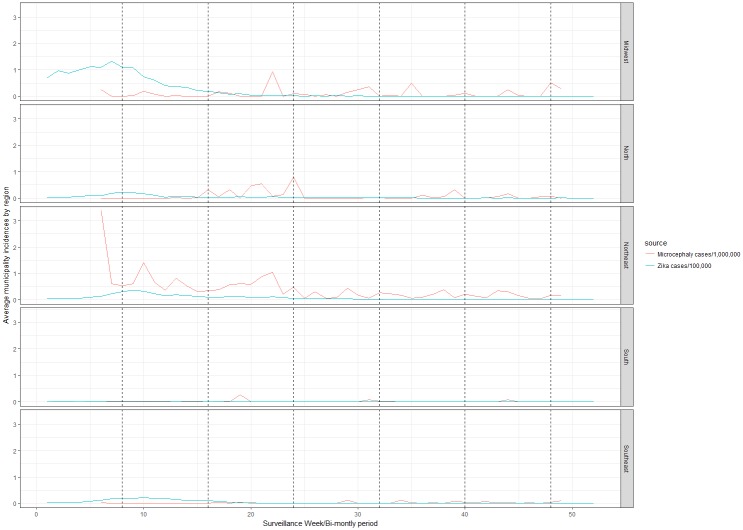
Bi-monthly Zika incidence and microcephaly incidence, by region in 2016, Brazil.

For both the first and second bi-monthly periods, the Northeast had its highest mean microcephaly incidence rates of 0.66 and 0.71, respectively. During the third and fourth bi-monthly periods, the density of microcephaly incidence clusters in the Northeast diminished. During the fifth and sixth bi-monthly periods, the Northeast had the highest mean microcephaly incidence rates of 0.20 and 0.18, respectively, remaining the region most affected across the observed period (Fig 2).

**Fig 3:**
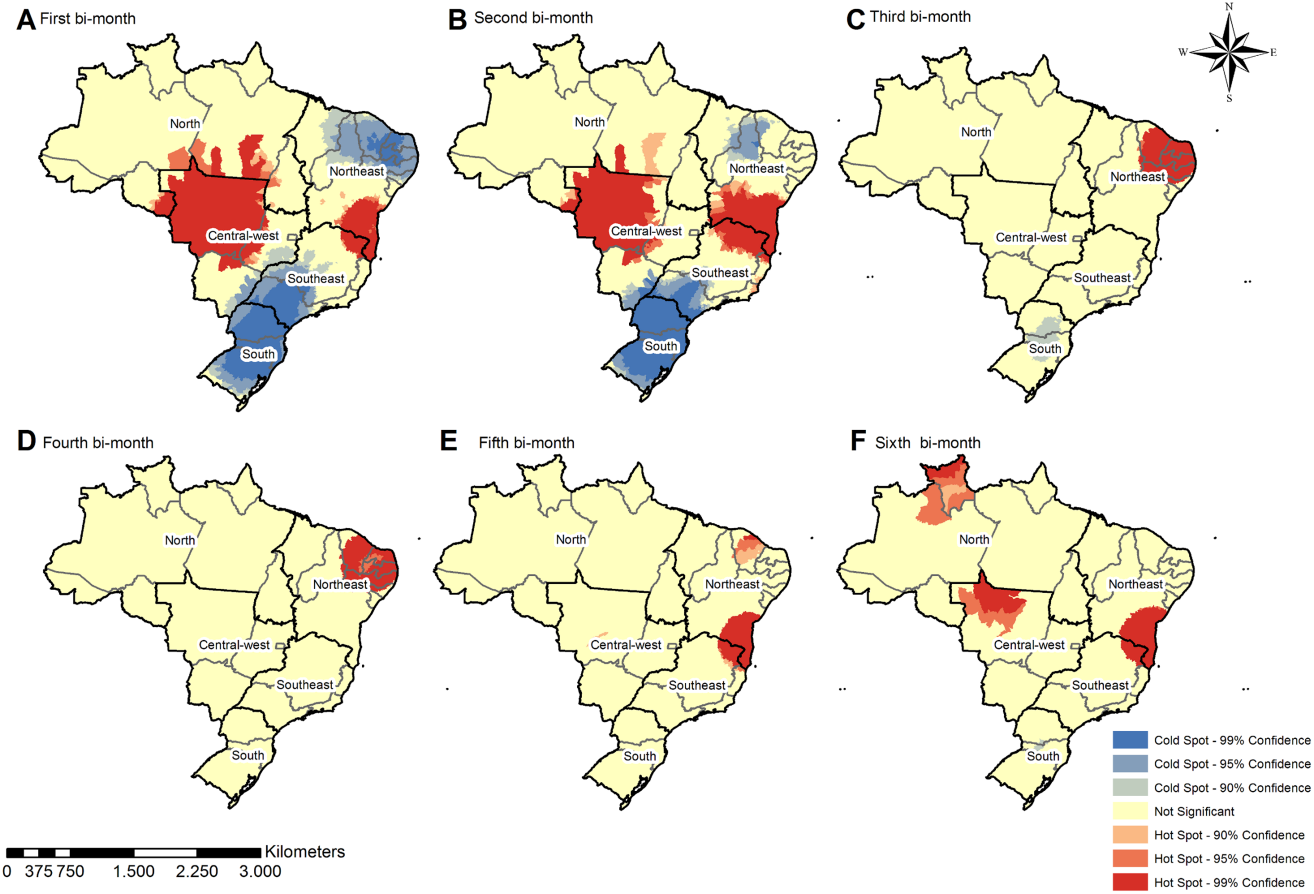
Spatial clusters—Getis Ord-Gi of Zika incidence by bi-monthly period, considering the 5570 Brazilian municipalities.

**Fig 4:**
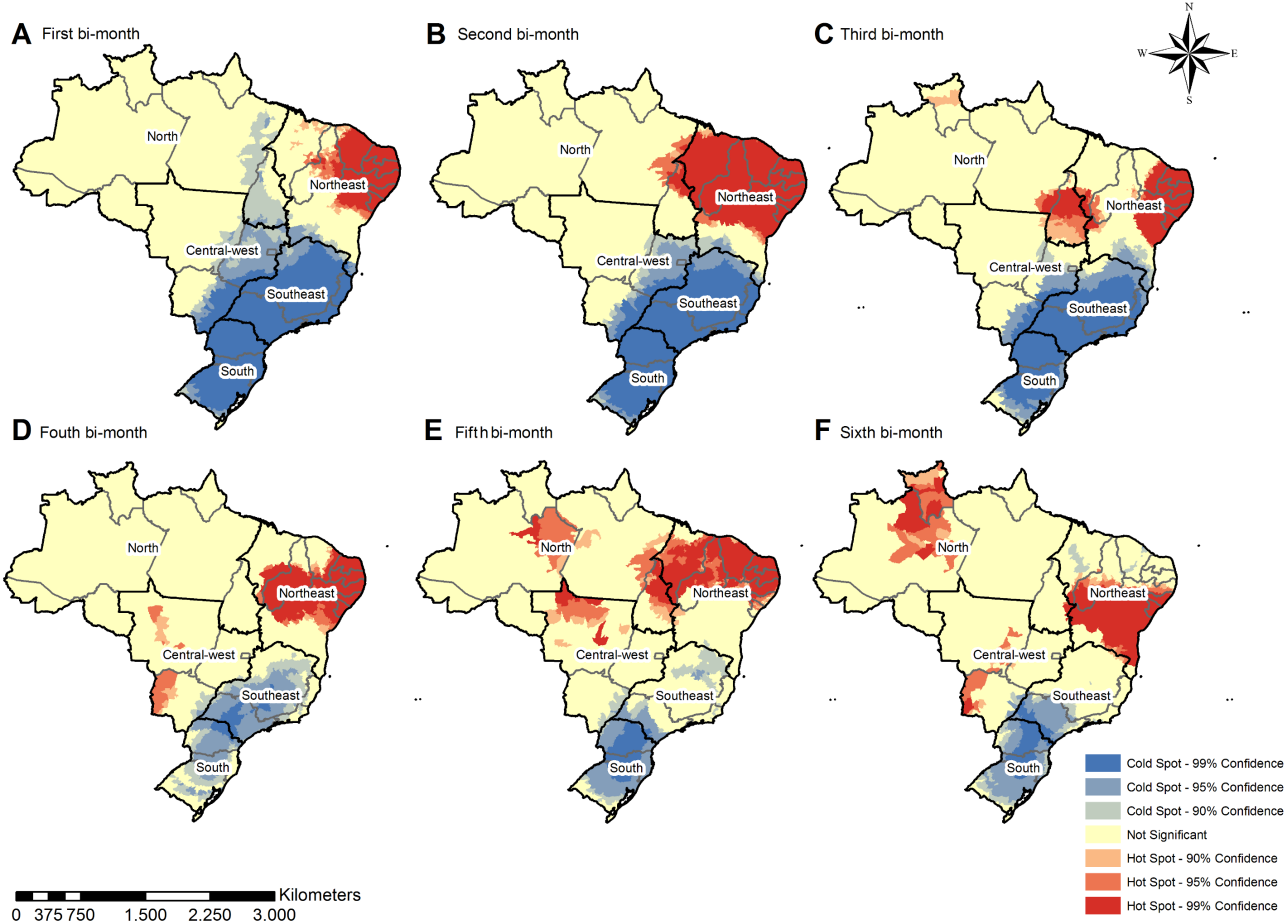
Spatial clusters—Getis Ord-Gi of microcephaly incidence by bi-monthly period, considering the 5570 Brazilian municipalities.

The geospatial distribution becomes more diffuse over time, with scattered groups of municipalities with high incidence in the Midwest, North, Northeast, and Southeast regions. Patterns of microcephaly geospatial distribution, distinct from that of ZIKV infection, tended to be concentrated in the Northeast during the first, second, and third bi-monthly periods (Figs 3 and 4). The results of the cluster analysis (Getis-Ord-Gi) highlighted ZIKV hotspots in the Midwest, Northeast, and Southeast regions during the first two bi-monthly periods; hotspots then shifted to the Northeast for the third and fourth bi-monthly periods. The fifth and sixth bi-monthly periods are marked by persisting hotspots in the Northeast, in addition to appearance of hotspots in the North, and the reemergence of those in the Midwest. In contrast to the varied locations of ZIKV incidence hotspots, those for microcephaly incidence varied less between the regions across all bi-monthly periods. From the third until sixth bi-monthly period, hotspots also appeared in the Midwest and North. The South and Southeast regions also both consistently remained coldspots of confirmed microcephaly across all bi-monthly periods (Figs 3 and 4).

**Fig 5:**
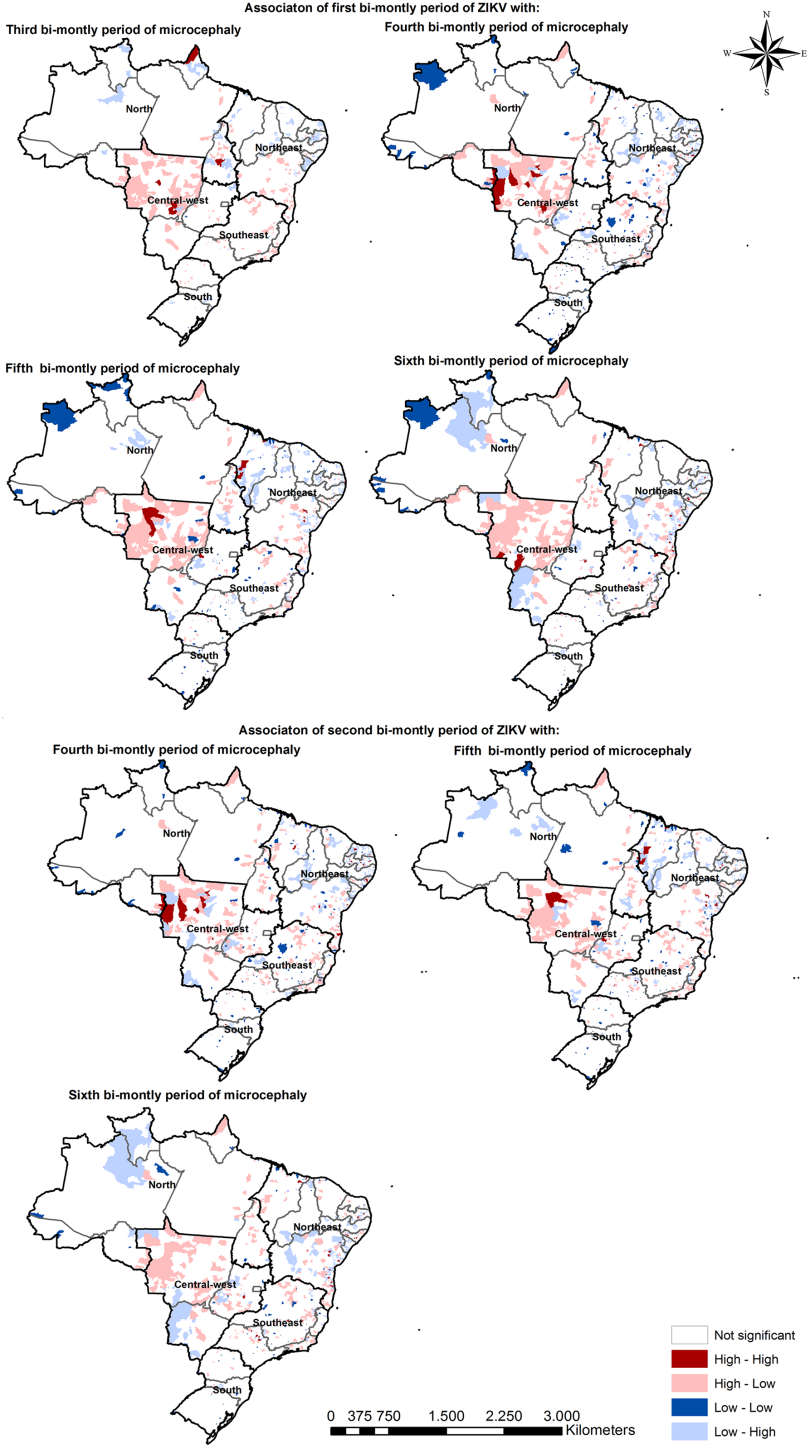
Spatial relation between the spread patterns of ZIKV and microcephaly, Bivariate Local Moran’s I analysis.

Bivariate Local Moran’s I analysis was performed, focusing on evidence of a possible spatial relation between the spread pattern of ZIKV and microcephaly (Fig 5). Considering the multiple time lag intervals adopted in the analysis, it was possible to identify an increasing wave in High ZIKV cluster areas becoming microcephaly High cluster areas across time. We are more interested in the High-Low/Low-High clusters in this representation. High-Low clusters (light red) represent areas with a high incidence of ZIKV surrounded by areas with low incidence of microcephaly, while Low-High areas are the inverse. From the results depicted in Fig 5, we noticed High-Low clusters mostly in the Midwest region. These clusters highlight regions with high incidence of ZIKV and low values of microcephaly, considering the different time lags observed. Analyzing the simultaneous presence of High-High clusters in the Midwest region, the High-Low clusters (light red) have the potential to become High-High clusters.

This finding highlights a relationship between an increase in ZIKV infection with a growth in microcephaly cases after two bi-monthly periods. Early in 2016, the ZIKV epidemic was already decreasing in the Northeast region, but was followed up with an increase in microcephaly (Low-High clusters). However, as we entered the 2016 epidemic year, the High clusters of ZIKV transitioned to the Midwest and North regions. The maps showing the association between the first bi-monthly period of ZIKV and the fifth and sixth bi-monthly periods of microcephaly demonstrate an increase of Low-High clusters in the Midwest and North regions, indicating that the microcephaly epidemic followed the distribution of the ZIKV infection. Additional analysis considering other bi-monthly periods as a starting point highlighted a similar growth pattern since a time lag of at least two months was observed (Fig 5).

## Discussion

From 2015–2016, Brazil experienced an unprecedented epidemic of microcephaly that carried devastating social and economic costs. A better understanding of the association between ZIKV and microcephaly was necessary to prevent a pandemic. Despite the importance and relevance of health geography, there is a lack of literature employing geospatial methods to analyze ZIKV and microcephaly. This study is the first to conduct a spatial-temporal evaluation of the association between ZIKV and microcephaly. Through this new approach, it was possible to identify evidence of an increase in microcephaly incidence associated with ZIKV incidence in the Midwest region of Brazil.

A potential link between ZIKV and microcephaly was first examined following reports of an abnormal rise in microcephaly incidence in Brazil’s Northeast region. This unexplained rise in microcephaly rates led public health authorities to begin epidemiological investigations. It was not until mid-2015 that suspicions regarding a link to ZIKV surfaced. This late identification of a possible cause carried implications for epidemiological surveillance. For most of 2015, there was no attention given to the causal link between ZIKV and microcephaly and, as a result, no reliable registry of ZIKV incidence rates was maintained. ZIKV was only designated as a disease of compulsory notification on February 17, 2016 [27]. Scientific evidence in support of the causal link later emerged in the beginning of 2016 [2,35,36]. In this context, it was not possible to analyze the spatial relationship between ZIKV and microcephaly in the early stages of the outbreak in the Northeast region. There were no data about ZIKV incidence before the 2016 microcephaly epidemic. After ZIKV was classified as a compulsory notification disease, more resources were invested toward more thorough and reliable reporting. Thus, data for all of 2016 is available at the municipal level. These improvements in disease surveillance facilitated research on the association between ZIKV and microcephaly spread patterns to identify those areas that are disproportionately affected and remain at an elevated risk. Our study contributes to these efforts and found a significant spatial pattern of association between both diseases.

The spread of ZIKV showed higher rates of infections in the Midwest in early 2016 that diminished by the end of the year. Confirmed ZIKV patterns in the Northeast and Southeast are consistent with a previous study of the spatial distribution of dengue fever in Brazil from 2014, one year before the Zika outbreak [37]. The Southeast, North, and Midwest regions experienced an increase in microcephaly incidence across 2016. This trend is explained by the high incidence of ZIKV previously in those regions. Our findings reveal that ZIKV incidence is positively associated with an increase in microcephaly incidence in the same location. Spread patterns of ZIKV and microcephaly cases in Brazil in 2016 suggest that a high number of cases of ZIKV in the Midwest are associated with a high number of cases of microcephaly in the region after a certain time lag. Measuring this association assists in probabilistic forecasting; monitoring the incidence of ZIKV may help predict where there will be increased incidence of microcephaly.

Our data support that the greatest burden of microcephaly could shift from the Northeast to other regions that reported a high volume of ZIKV during the beginning of 2016. A similar finding was reported by De Oliveira et al [29]. Our findings are of importance to health care providers and managers in these regions who should anticipate a greater need for prenatal care and adjust protocols in light of new systematic public health data about both diseases. The establishment of a regular monitoring system informed by the methodologies defined in the present study is needed to further confirm if observed relationships are maintained over time.

Best practices for pregnancy management during the ZIKV epidemic detail clinical manifestations of infection, endorse serological testing [38] depending on symptom presentation and timing of acute infection, and recommend routine ultrasounds before 24 weeks gestation [35]. Laboratory confirmation of ZIKV facilitates systematic efforts to estimate its prevalence and risk [39]. These practices need to be considered in the Midwest region for pregnancy management, as our findings suggest that this region will face an increase in microcephaly cases. Therefore, training actions for primary care professionals are recommended, as well as a revision of protocols related to pregnant women in areas at risk.

The transmission of ZIKV and other arbovirus diseases through genus *Aedes* mosquitos places every region with a tropical climate in a position of risk. The European Centre for Disease Prevention and Control mapped and categorized patterns of ZIKV transmission globally [40]. Several countries in South and Central America, Africa, and portions of Oceania were categorized by the World Health Organization as regions with active circulation of ZIKV. Tracking the relationship and behavior of ZIKV and microcephaly geographically is essential to design and implement response strategies to avoid outbreaks of microcephaly and other neurological complications [41].

The observed reduction in ZIKV incidence in the country as a whole, in fact, requires additional explanation. The incidence pattern of ZIKV followed the same tendency of dengue and chikungunya in 2016; there were peaks in incidence during the first months of the year followed by a decrease [8]. This trend might be due to the rainy season in Brazil that lasted from November until the end of March for most of the country. The increase in the rainfall index contributes to the growth of *Aedes* mosquito breeding sites, producing a rise in diseases transmitted through this vector. Brazil has continental dimensions with different climatic characteristics between its regions, as well as historical regional inequalities related to access to basic sanitation services. Simultaneous access to water supply by general network, sanitary sewage by general or rainwater network, and direct or indirect collection of garbage are still unequal among the five Brazilian regions. Municipalities lacking adequate sanitation are subject to a higher risk of infestation by *Aedes* and are consequently exposed to a higher risk of dengue, chikungunya, and ZIKV [42]. Even with this hypothesis, there is not yet longitudinal data on ZIKV to attribute the decrease in incidence rates to seasonal events like the rainy season. Additional hypotheses are being tested to explain the decline in ZIKV cases, including a massive infection perspective leading to a lack of susceptible individuals [43].

As limitations of the present study, we can highlight the lack of laboratory confirmation for part of the cases considered. However, they meet the epidemiological case criteria. Another limitation was the impossibility of estimating the incidence rates of ZIKV infection only in women due to the absence of the gender variable in the database. Thus, the estimates refer to the overall rates including men and women. The presence of gender information, as well as other details, such as pregnancy status or week of pregnancy, could increase the surveillance capabilities of the present information. Additionally, this information carries the potential to better support the relationship among ZIKV and microcephaly. However, as the current evidence supports sexual transmission [38,44–47] and salivary transmission [48], a high incidence in men increases the chances of infection in women. Thus, the overall incidence is a good estimator of the disease in the population. The fact that compulsory notification was only instituted in Brazilian health services in February 2016 may have led to an underreporting of both events (ZIKV and microcephaly), but especially of the first. The detection of abnormal levels of microcephaly cases was the trigger event responsible for raising additional investigations. Only after several months was a clear relation between microcephaly and ZIKV established. Thus, ZIKV was not on the surveillance radar of Brazilian epidemiological authorities when the microcephaly cases peaked. Therefore, there is no solid information about ZIKV incidence before the first rise in microcephaly cases, limiting the possibility of additional investigations regarding the first outbreak of microcephaly. As a consequence, there was potential bias towards the null hypothesis in association estimates, i.e., if all cases of ZIKV infection had been effectively reported, the associations found would have been even stronger.

Our study helps clarify the spatial association of microcephaly incidence in neonates whose mothers were previously infected with ZIKV. However, doubts remain about a possible relationship between the time of infection in pregnancy and the severity of sequelae in the fetus, or whether the symptoms of microcephaly depend on virus titers in fluids but not at the time of infection [49]. We don’t know if co-infections like dengue and chikungunya play any role in the severity of microcephaly [50]. Little is known [51,52] about the consequences of co-infection events [50,53]. What are the mechanisms used to break placenta barriers? What cells are involved in the pathogenesis of severe disease? [54] It is imperative to establish *Aedes aegypti* control in the Americas and the rest of the world to prevent the spread of ZIKV to new areas [55]. Understanding these and other issues may contribute to plans to control new outbreaks of this or other variations of the virus.
